# Evidence mapping on how to perform an optimal surgical repair of large hiatal hernias

**DOI:** 10.1007/s00423-023-03190-y

**Published:** 2023-12-21

**Authors:** Felix Nickel, Philip C. Müller, Amila Cizmic, Frida Häberle, Markus K. Muller, Adrian T. Billeter, Georg R. Linke, Oliver Mann, Thilo Hackert, Christian A. Gutschow, Beat P. Müller-Stich

**Affiliations:** 1 Department of General, Visceral and Thoracic Surgery, University Medical Center Hamburg-Eppendorf, Martinistraße 52, 20246 Hamburg, Germany; 2 Department of General, Visceral and Transplant Surgery, Heidelberg University Hospital, Heidelberg, Germany; 3 Department of Visceral and Transplant Surgery, University Hospital Zurich, Zurich, Switzerland; 4 Department of Surgery, Cantonal Hospital Thurgau, Frauenfeld, Switzerland; 5 Department of Digestive Surgery, University Digestive Healthcare Center Basel, Basel, Switzerland; 6 Department of Surgery, Hospital STS Thun AG, Thun, Switzerland

**Keywords:** Hiatal hernia, Fundoplication, Cruroplasty

## Abstract

**Background:**

Symptomatic and large hiatal hernia (HH) is a common disorder requiring surgical management. However, there is a lack of systematic, evidence-based recommendations summarizing recent reviews on surgical treatment of symptomatic HH. Therefore, this systematic review aimed to create evidence mapping on the key technical issues of HH repair based on the highest available evidence.

**Methods:**

A systematic review identified studies on eight key issues of large symptomatic HH repair. The literature was screened for the highest level of evidence (LE from level 1 to 5) according to the Oxford Center for evidence-based medicine’s scale. For each topic, only studies of the highest available level of evidence were considered.

**Results:**

Out of the 28.783 studies matching the keyword algorithm, 47 were considered. The following recommendations could be deduced: minimally invasive surgery is the recommended approach (LE 1a); a complete hernia sac dissection should be considered (LE 3b); extensive division of short gastric vessels cannot be recommended; however, limited dissection of the most upper vessels may be helpful for a floppy fundoplication (LE 1a); vagus nerve should be preserved (LE 3b); a dorso-ventral cruroplasty is recommended (LE 1b); routine fundoplication should be considered to prevent postoperative gastroesophageal reflux (LE 2b); posterior partial fundoplication should be favored over other forms of fundoplication (LE 1a); mesh augmentation is indicated in large HH with paraesophageal involvement (LE 1a).

**Conclusion:**

The current evidence mapping is a reasonable instrument based on the best evidence available to guide surgeons in determining optimal symptomatic and large HH repair.

**Supplementary Information:**

The online version contains supplementary material available at 10.1007/s00423-023-03190-y.

## Introduction

Hiatal hernia (HH) is a common disorder characterized by a protrusion of abdominal organs through the esophageal hiatus into the thoracic cavity [1]. Currently, HH is classified into four types. Type I HH, the so-called sliding hernia, is the most common form. Surgical treatment for this type of hernia is only indicated when concomitant gastroesophageal reflux disease is present. However, due to the excellent results of medical reflux therapy, only a minority of these patients undergo surgical treatment. Type II–IV hernias are characterized by paraesophageal involvement. Surgical treatment is indicated in the presence of relevant symptoms, including pain, dysphagia, anemia, gastroesophageal reflux, and cardiopulmonary restrictions when other causes are ruled out. The risk of progression and associated complications, such as incarceration, should also be considered. The aim of surgical repair of symptomatic and large HH is the durable relief of symptoms by repositioning the hernia contents into the abdomen and repairing the esophageal hiatus. The introduction of minimally invasive surgery (MIS) in the early 1990s changed the approach to surgical HH repair [2]. Although several studies have evaluated different technical aspects during HH repair, a systematic summary of the available evidence was missing until the SAGES guidelines for the management of HH were published in 2013 [1]. Among other things, the guidelines reflected on the technical aspects such as the type of surgical access, the advantage of a hernia sac excision, and whether a routine mesh cruroplasty, routine fundoplication, and routine gastropexy should be recommended. However, the literature search used to establish the guidelines was conducted in 2011, and recent, updated, and systematic evidence-based reviews summarizing the latest recommendations for HH repair are lacking. A more recently published Delphi consensus has addressed current recommendations on managing paraesophageal HH based on European expert opinions [3].

Nevertheless, because recommendations on the key surgical steps are often based on expert opinion rather than up-to-date evidence, the aim of the present systematic review was to create evidence mapping on technical key steps of surgical repair of symptomatic and large HH based on the most recent, highest-level evidence available for each surgical step.

## Methods

This systematic review was conducted according to the PRISMA guidelines [4]. Article screening, data extraction, and critical appraisal were independently performed by three investigators (PCM, PP, AC). Disagreements were resolved by consulting the senior author of the study. No sponsors or commercial entities had any role in the study design, data collection, data analysis, data interpretation, or writing of the report.

### Literature search, study selection, and data collection

A comprehensive search of the electronic databases MEDLINE (via PubMed), Web of Science, and CENTRAL was carried out [5]. The references of the included studies were reviewed to locate further appropriate studies. The keyword algorithm used is depicted in Appendix S1 (supporting information). No restrictions regarding language or date of publication were set. The last search was performed on June 30, 2023.

### Key steps during HH repair

Important technical aspects and key issues during the surgical repair of symptomatic and large HH were defined before conducting the literature search. The following eight topics were considered:

1. Type of surgical access
2. Dissection of the hernia sac
3. Division of short gastric vessels
4. Preservation of the vagus nerves
5. Cruroplasty
6. Addition of a fundoplication
7. Type of fundoplication
8. Mesh augmentation

### Eligibility criteria

According to the levels defined by the Oxford Centre for Evidence-based Medicine (OCEBM Levels of Evidence Working Group, “The Oxford Levels of Evidence 2” (https://www.cebm.net/), literature was screened for the highest level of evidence, ranging from level 1 to 5 for each technical aspect addressed (see outcome categories). Only the highest available evidence was included, and whenever the level of evidence was considered sufficient, lower evidence-level studies were not considered further. Additional exclusion criteria included patients aged <18 years and animal studies.

### Outcome categories

Different outcome measures were considered according to the available evidence for each technical aspect. Data is presented according to the best evidence available for each topic.

#### Type of surgical access

The main outcome was postoperative complications as a surrogate marker for surgical trauma. Additional parameters were operative time, hospital stay, and reoperations.

#### Dissection of the hernia sac

HH recurrence was chosen as the main outcome. Conversions and postoperative complications were additional parameters.

#### Division of short gastric vessels

The main outcome was the reoperation rate as a surrogate for symptomatic recurrences and severe complications. Subordinate parameters were lower esophageal sphincter pressure, gas bloat, dysphagia, postoperative complications, and reflux recurrence.

#### Preservation of the vagus nerves

Outcomes measured were diarrhea, dumping syndrome, delayed gastric emptying, and gas bloating.

#### Cruroplasty

The main outcome parameters were dysphagia and the need for reoperation. Complimentary parameters were lower esophageal sphincter pressure, heartburn, and patient satisfaction.

#### Addition of a fundoplication

The main outcome was reflux as measured with esophageal 24h pH-metry. Further parameters were reflux syndrome score, dysphagia, esophagitis, postoperative complications, and quality of life.

#### Type of fundoplication

The main outcome was dysphagia. Additional parameters were postoperative heartburn, esophagitis, postoperative complications, and reoperations.

#### Mesh augmentation

The main outcome was the HH recurrence rate. Complimentary parameters were reoperation rate, complication rate, and mesh-associated complications.

### Critical appraisal

The included studies were critically appraised with the most appropriate instrument for each type, i.e., for systematic reviews, studies were compared to the methods used in the Cochrane handbook [Higgins, JPT. *Cochrane Handbook for Systematic Reviews of Interventions Version 5.1.0* (updated March 2011), The Cochrane Collaboration, 2011. Available from www.cochrane-handbook.org . (Accessed 01 Nov 2015)]. For RCTs, the Cochrane risk of bias assessment tool was used (Wieder Higgins). For non-randomized studies, the ACROBAT-NRSI tool [Sterne JAC, Higgins JPT, Reeves BC on behalf of The Development Group for ACROBAT-NRSI. A Cochrane Risk of Bias Assessment Tool for Non-Randomized Studies of Interventions (ACROBAT-NRSI), Version 1.0.0, Sept 24th, 2014)] was used.

## Results

**Fig. 1:**
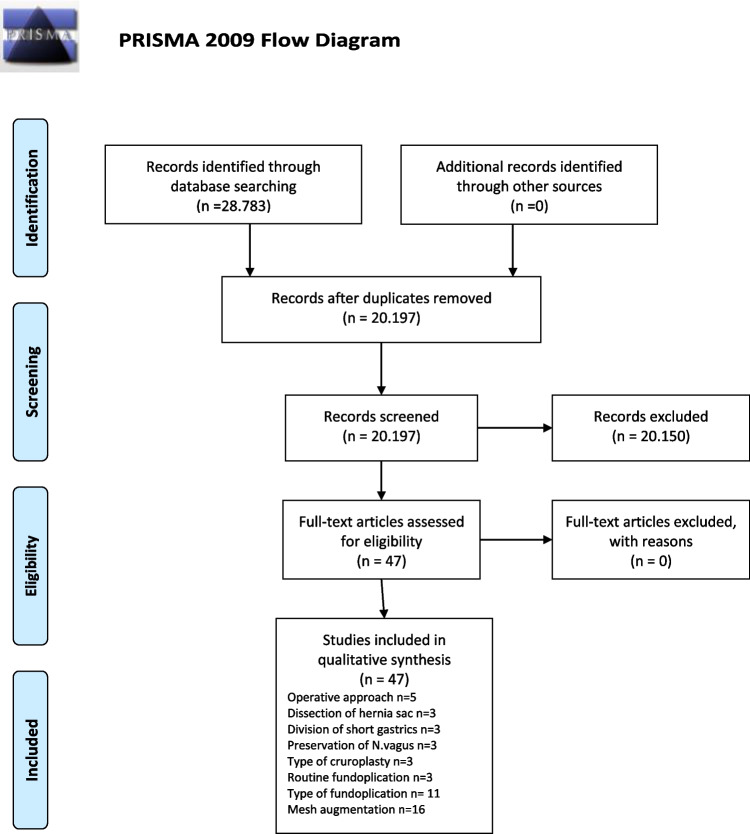
PRISMA Flow Chart of the Study

A total of 28.783 studies matching the keyword algorithm were found (Fig. 1). Forty-seven studies consisting of 17 meta-analyses of exclusively randomized controlled trials (RCTs), ten meta-analyses of RCTs and non-RCTs, 12 RCTs, four non-RCTs, one MA of non-RCTs, one systematic review of non-RCTs, and one retrospective study were included (Table 1).

**Table 1:** Characteristics of the included studies

	Type of study	Year	Country	*N*	Intervention	Control
Type of operative approach						
Peters et al.	MA-RCT	2009	Australia	1036	Lap. anti-reflux surgery	Open anti-reflux surgery
Qu et al.	MA-RCT	2014	China	1067	Lap. anti-reflux surgery	Open anti-reflux surgery
Wilhelm et al.	Prospective clinical trial	2021	Switzerland	55	Robotic-assisted large hiatal hernia repair	Lap. large hiatal hernia repair
Lang et al.	RCT	2022	Germany	40	Robotic-assisted anti-reflux surgery	Lap. anti-reflux surgery
Ma et al.	MA-Non RCT	2023	China	10,078	Robotic-assisted hiatal hernia repair	Lap. hiatal hernia repair
Dissection of the hernia sac						
Edye et al.	Non-RCT	1997	USA	55	Lap. hiatal hernia repair with excision of the hernia sac	Lap. hiatal hernia repair without excision of the hernia sac
Watson et al.	Non-RCT	1999	Australia	68	Lap. hiatal hernia repair with dissection of the hernia sac	Lap. hiatal hernia repair without dissection of the hernia sac
Wu et al.	Non-RCT	1998	USA	38	Lap. hiatal hernia repair with excision of the hernia sac	Lap. hiatal hernia repair without excision of the hernia sac
Division of short gastric vessels						
Markar et al.	MA- RCT	2011	UK	388	Lap. Nissen fundoplication with a division of short gastric vessels	Lap. Nissen fundoplication without division of short gastric vessels
Khatri et al.	MA-RCT	2011	UK	388	Lap. Nissen fundoplication with a division of short gastric vessels	Lap. Nissen fundoplication without division of short gastric vessels
Kinsey-Trotman et al.	RCT	2018	Australia	102	Lap. Nissen fundoplication with a division of short gastric vessels	Lap. Nissen fundoplication without division of short gastric vessels
Preservation of the vagus nerve						
Oelschlager et al.	Retrospective study	2008	USA	102	Reoperative antireflux surgery of paraesophageal hernia repair with vagotomy	Reoperative antireflux surgery of paraesophageal hernia repair without vagotomy
Ozdogan et al.	RCT	2013	Turkey	40	Fundoplication with dissection of the hepatic branch of the anterior vagus nerve	Fundoplication without dissection of the hepatic branch of the anterior vagus nerve
Van Rijn et al.	SR Non-RCT	2016	Netherlands	893	Antireflux surgery with dissection of vagus nerves	Antireflux surgery without dissection of vagus nerves
Cruroplasty						
Watson et al.	RCT	2001	Australia	102	Lap. Nissen fundoplication with anterior hiatal repair	Lap. Nissen fundoplication with posterior hiatal repair
Wijnhoven et al.	RCT	2008	Australia	102	Lap. Nissen fundoplication with anterior hiatal repair	Lap. Nissen fundoplication with posterior hiatal repair
Chew et al.	RCT	2011	Australia	102	Lap. Nissen fundoplication with anterior hiatal repair	Lap. Nissen fundoplication with posterior hiatal repair
Addition of a fundoplication						
Müller-Stich et al.	RCT	2015	Germany	40	Lap. mesh-augmented hiatoplasty with cardiophrenicopexy	Lap. mesh-augmented hiatoplasty with fundoplication
Li et al.	RCT	2019	China	122	Lap. hiatal hernia repair without fundoplication	Lap. hiatal hernia repair with Nissen fundoplication
Clapp et al.	MA-RCT/Non-RCT	2022	USA	8600	Lap. hiatal hernia repair without fundoplication	Lap. hiatal hernia repair with fundoplication
Type of fundoplication						
Broeders et al.	MA-RCT	2010	Netherlands	792	Lap. partial posterior (270°) fundoplication	Lap. total (360°) fundoplication
Tan et al.	MA-RCT	2010	China	939	Lap. partial posterior (270°) fundoplication	Lap. total (360°) fundoplication
Broeders et al.	MA-RCT	2011	Netherlands	683	Lap. anterior (90°, 120°, 180°) fundoplication	Lap. partial posterior (180°, 200°, 360°) fundoplication
Ma et al.	MA-RCT	2011	China	1374	Lap. partial (90°, 180°, 200°, 270°, 300°) fundoplication	Lap. total (360°) fundoplication
Broeders et al.	MA-RCT	2013	Netherlands	458	Lap. partial anterior (180°) fundoplication	Lap. total 360°) fundoplication
Memon et al.	MA-RCT	2014	Australia	840	Lap. anterior (90°, 120°, 180°) fundoplication	Lap. posterior (270°-360°) fundoplication
Tian et al.	MA-RCT	2015	China	1564	Lap. partial posterior (270°) fundoplication	Lap. total (360°) fundoplication
Du et al.	MA-RCT	2016	China	1192	Lap. partial posterior (270°) fundoplication	Lap. total (360°) fundoplication
Hopkins et al.	RCT	2020	Australia	191	Lap. partial anterior (90°) fundoplication	Lap. total (360°) fundoplication
Analatos et al.	RCT	2022	Sweden	70	Lap. total (360°) fundoplication	Lap. partial posterior partial (200°) fundoplication
Tasoudis et al.	MA-RCT/Non-RCT	2023	USA	2774	Lap. total (360°) fundoplication	Transthoracic Berkley Mark IV fundoplication
Mesh augmentation						
Antoniou et al.	MA-RCT	2012	Germany	267	Large hiatal hernias with prosthetic herniorrhaphy	Large hiatal hernias with suture cruroplasty
Antoniou et al.	MA-RCT/Non-RCT	2015	Germany	295	Hiatal hernia repair with biological mesh	Hiatal hernia repair with suture cruroplasty
Müller-Stich et al.	MA-RCT/Non-RCT	2015	Germany	5499	Hiatal hernias with prosthetic herniorrhaphy	Hiatal hernias without prosthetic herniorrhaphy
Tam et al.	MA-RCT/Non-RCT	2015	USA	1194	Large hiatal hernias with prosthetic herniorrhaphy	Large hiatal hernias with suture cruroplasty
Zhang et al.	MA-RCT/Non-RCT	2017	China	1474	Hiatal hernias with prosthetic herniorrhaphy	Hiatal hernias without prosthetic herniorrhaphy
Ilyashenko et al.	RCT	2018	Ukraine	98	Large hiatal hernia repair with mesh	Large hiatal hernia repair with suture cruroplasty
Oor et al.	RCT	2018	Netherlands	72	Hiatal hernia repair with non-absorbable suture	Hiatal hernia repair with sutures reinforced with non-absorbable mesh
Campos et al.	MA-RCT/Non-RCT	2019	Brazil	520	Hiatal hernia repair with mesh reinforcement	Hiatal hernia repair with primary suture
Memon et al.	MA-RCT	2019	Australia	478	Large hiatal hernias with prosthetic herniorrhaphy	Large hiatal hernias with suture cruroplasty
Sathasivam et al.	MA-RCT/Non-RCT	2019	UK	942	Large hiatal hernias with prosthetic herniorrhaphy	Large hiatal hernias with suture cruroplasty
Watson et al.	RCT	2020	Australia	126	Large hiatal hernias with suture	Large hiatal hernia repair with absorbable and non-absorbable mesh reinforcement
Angeramo et al.	MA-RCT	2022	Argentina	735	Hiatal hernia repair with mesh reinforcement	Hiatal hernia repair with primary suture
Petric et al.	MA-RCT	2022	Australia	762	Hiatal hernias with suture	Hiatal hernias with mesh
Temperly et al.	MA-RCT	2022	Ireland	766	Hiatal hernia repair with suture cruroplasty	Hiatal hernia repair with absorbable and non-absorbable mesh reinforcement
Clapp et al.	MA-RCT/non-RCT	2023	USA	1580	Hiatal hernia repair with biological mesh	Hiatal hernia repair with suture cruroplasty
Rajkomar et al.	MA-RCT/Non-RCT	2023	UK	1670	Large hiatal hernias with suture cruroplasty	Large hiatal hernia repair with mesh

*N*, number of participants; *MA-RCT*, meta-analysis of randomized controlled trials; *lap*., laparoscopic; *Non-RCT*, not randomized controlled clinical trials; *RCT*, randomized controlled trial; *SR non-RCT*, systematic review of non-randomized controlled trials; *MA RCT/Non-RCT*, meta-analysis of randomized and non-randomized controlled trials

## Type of surgical approach

Two meta-analyses of RCTs comparing minimally invasive with open antireflux surgery were available, but no studies compared open and minimally invasive approaches for symptomatic and large HH repair [6, 7]. The meta-analysis by Peters et al. included twelve RCTs (one on pediatric patients) with 503 patients undergoing open and 533 patients undergoing minimally invasive antireflux surgery. The minimally invasive approach reduced postoperative morbidity [odds ratio (OR): 0.35, 95% confidence interval (CI) 0.16–0.75; *P *= 0.007)], while the operative time was 39 min longer [weighted mean difference (WMD): 39.03 95%CI: 17.99–60.05; *P* < 0.001)]. In the minimally invasive group, the hospital stay was reduced by a mean of 2.7 days (WMD: −2.68, 95%CI −3.54 to −1.81; *P* < 0.0001). Of concern was an increased reoperation rate for symptomatic recurrences in the minimally invasive group (OR 1.79, 95%CI 1.00–3.22; *P *= 0.05). However, the study did not compare the reoperation rate for all indications [6]. Compared to the analysis by Peters et al. the most recent meta-analysis included two new RCTs, while two previous studies, one of which focused on pediatric patients, were excluded. In the meta-analysis by Qu et al. both short-term postoperative morbidity (10.2% vs. 26.4%; OR 0.31, 95%CI 0.17–0.56; *P* < 0.001) and long-term complications (2.1% vs. 10.9%; OR 0.24, 95%CI 0.07–0.80; *P *= 0.02) were reduced following a minimally invasive approach. As in the previous study, the operation time was prolonged for the minimally invasive approach [mean difference (MD) 32.5 min, 95%CI 13.54–51.55 min; *P* < 0.001)], and the hospital stay was two days shorter (MD −2.04 days, 95%CI −2.95 to −1.12 days; *P* < 0.001). There was no difference in the reoperation rate, including symptomatic recurrences and other indications such as incisional hernia repair (10.3 % vs. 14.1 %; OR 0.75, 95%CI 0.27–2.06; *P *= 0.57) [7]. There was a low risk of bias as the two included meta-analyses clearly stated the addressed main question, were unlikely to have missed important studies, used appropriate criteria to select the studies included, and made sure that these were sufficiently valid and had results that were similar to each other (Table 2).

**Table 2:** Risk of bias assessed for the included systematic reviews

	What question did the systematic review address?	Is it unlikely that important, relevant studies were missed?	Were the criteria used to select articles for inclusion appropriate?	Were the included studies sufficiently valid for the type of question asked?	Were the results similar from study to study?
Peters 2009	Yes	Yes	Yes	Yes	Yes
Broeders 2010	Yes	Yes	Yes	Yes	Yes
Ma 2010	Yes	Yes	Yes	Yes	Yes
Tan 2010	Yes	Yes	Yes	Yes	Yes
Broeders 2011	Yes	Yes	Yes	Yes	Yes
Khatri 2011	Yes	No	Yes	Yes	Yes
Markar 2011	Yes	Yes	Yes	Yes	Yes
Antoniou 2012	Yes	Yes	Yes	Yes	Yes
Broeders 2013	Yes	Yes	Yes	Yes	Yes
Memon 2014	Yes	Yes	Yes	Yes	Yes
Qu 2014	Yes	Yes	Yes	Yes	Yes
Antoniou 2015	Yes	Yes	Yes	Yes	Yes
Müller-Stich 2015	Yes	Yes	Yes	Yes	Yes
Tam 2015	Yes	Yes	Yes	Yes	Yes
Tian 2015	Yes	Yes	Yes	No	Yes
Du 2016	Yes	Yes	Yes	Yes	Yes
Van Rijn 2016	Yes	Yes	Yes	Yes	No
Zhang 2017	Yes	Yes	Yes	Yes	Yes
Campos 2019	Yes	Yes	Yes	Yes	No
Memon 2019	Yes	Yes	Yes	Yes	Yes
Sathasivam 2019	Yes	No	Yes	Yes	Yes
Angeramo 2022	Yes	Yes	Yes	No	Yes
Clapp 2022	Yes	Yes	Yes	Yes	Yes
Petric 2022	Yes	Yes	Yes	Yes	Yes
Temperly 2022	Yes	Yes	Yes	Yes	Yes
Clapp 2023	Yes	Yes	Yes	Yes	Yes
Ma 2023	Yes	Yes	Yes	Yes	No
Rajkomar 2023	Yes	Yes	Yes	Yes	Yes
Tasoudis 2023	Yes	Yes	Yes	Yes	Yes

More recently, it has been reported that several studies have reported that robotic-assisted hiatal hernia repair offers enhanced precision and improved visualization [8–10]. There were no RCTs comparing open or laparoscopic approaches to robotic-assisted approaches in large hiatal hernia repair. However, there was one meta-analysis identified that compared laparoscopic with robotic-assisted hiatal hernia repair. A MA by Ma et al. included seven studies (four retrospective and three database studies) with 10,078 patients [11]. The postoperative complication rate was 4.25% (302/7111) for the laparoscopic and 3.49% (38/1088) for the robotic-assisted approach (OR 0.52; 95% CI 0.36 to 0.75, *P* < 0.001). Other measured parameters, such as intraoperative complications, operative time, and 30-day readmission, showed no difference between both groups. A prospective comparative single-center study by Wilhelm et al. compared the robotic-assisted with laparoscopic repair of a complete upside-down stomach hiatal hernia (the RATHER study) [12]. The study included 36 robotic-assisted and 19 laparoscopic hiatal hernia repairs of upside-down stomach. Although the median operative time in the robotic-assisted approach was more prolonged than in laparoscopic (232 vs. 163 min., *P* < 0.001), the mortality and morbidity rate was comparable between the two groups.

An RCT by Lang et al. reported 12-year follow-ups on patients undergoing robotic-assisted versus laparoscopic anti-reflux surgery [13]. The authors reported on the ROLAF RCT, which included 40 patients randomized into robotic-assisted (*n *= 20) and laparoscopic (*n *= 20) groups. The 12-year follow-up showed no difference between the two groups regarding postoperative symptoms, quality of life, and treatment failure. Similar results were reported after the study initially and after a 1-year follow-up [14, 15]. There is insufficient scientific evidence to confirm the benefit of either of these two surgical approaches over the other. A recent MA by Huttman et al. comprised RCTs and clinical studies comparing robotic-assisted versus laparoscopic anti-reflux surgery reporting standard [16]. The authors reported the heterogenous outcomes reporting, emphasizing that not a single outcome was reported in all 23 included RCTs. This makes a genuine comparison of the two surgical approaches complex and points to the utility of unified reporting standards.

### Conclusion

MIS offers several benefits over the open approach, such as reduced postoperative morbidity and shorter hospital stay. Although it has risen in popularity and as a research subject, the robotic-assisted approach provides little benefits compared to conventional laparoscopic large hiatal hernia repair.

### Recommendation (Level of evidence 1a)

The minimally invasive approach is recommended for symptomatic and large HH repair.

## Dissection of the hernia sac

Three retrospective case series comparing complete with incomplete hernia sac dissection were available. Edye et al. compared 55 patients with paraesophageal HH with a median follow-up of 29 months. Five early recurrences occurred in the 25 patients with incomplete resection of the hernia sac. Two patients that needed conversion were excluded from the analysis. No early recurrences were observed in the remaining 30 patients who had a complete excision (*P *= 0.015), and one patient was excluded from the analysis due to conversion. Other than early recurrence, no differences in postoperative complications (16% vs. 13%; *P *= 1.0) were found [17]. A study by Watson et al. compared 46 patients with a complete HH sac dissection to 40 patients without. The median follow-up was 2 years. While only one early recurrence was reported in each group, a 40% conversion rate in patients with incomplete hernia sac dissection was observed compared to 9% in patients with a complete dissection (*P* < 0.001). Furthermore, postoperative complications were reduced when the hernia sac was dissected (20% vs. 6%; *P *= 0.103) [18]. Finally, Wu et al. compared 25 patients with hernia sac excision to 12 patients without. The median follow-up was up to 1 year. Thirty-day morbidity (12% vs. 25%; *P *= 0.366) and early recurrence (16% vs. 33%; *P *= 0.394) were reduced, albeit not significantly [19]. Regarding bias risk, the overall risk of bias for Watson et al. was low. Because Edye et al. and Wu et al. did not report on homogenous intervention groups, their outcomes were not systematically evaluated, and no systematic follow-up was performed, their measurements of interventions and outcomes hold a moderate risk of bias (Table 3). Furthermore, those studies did not report the results of the whole study population, resulting in a moderate risk of bias in the selection of reported results, as well. The overall risk of bias for the studies by Edye et al. and Wu et al. was therefore graded as moderate (Table 4). One of the potential complications during hiatal sac dissection is the occurrence of the pleural lesion, which can result in pneumothorax. Edye et al. and Watson et al. did not report this complication, while Wu et al. reported no pleural lesion in their study.

**Table 3:** Risk of Bias assessed for non-randomized studies

	Bias due to confounding	Bias in the Selection of participants	Bias in measurement of interventions	Bias due to departures from intended interventions	Bias due to missing data	Bias in measurement of outcomes	Bias in the selection of reported results	Overall RoB judgment
Edye et al.	Low	Low	Moderate	Low	No information	Moderate	Moderate	Moderate
Watson et al.	Low	Low	Low	Low	Low	Low	Low	Low
Wilhelm et al.	Low	Low	Moderate	Low	Moderate	Low	Moderate	Moderate
Wu et al.	Moderate	Low	Moderate	Low	Serious	Moderate	Moderate	Moderate
Oelschlager et al.	Moderate	High	Moderate	Moderate	High	Low	Low	Moderate

### Conclusion

Complete dissection and excision of the hernia sac may reduce the risk of conversion, postoperative recurrences, and complications, maybe due to better visualization and mobilization of the esophagus and a tension-free positioning of the stomach within the abdominal cavity.

### Recommendation (Level of evidence 3b)

A complete dissection of the hernia sac should be performed whenever possible.

## Division of short gastric vessels

No studies were found comparing the surgical treatment of HH regarding the choice of performing a division of short gastric vessels. Thus, studies that included patients undergoing antireflux surgery with or without the division of short gastric vessels were evaluated. Two separate meta-analyses examined the same five RCTs investigating the effect of dividing the short gastric vessels in antireflux surgery [20, 21]. No differences in reoperations (OR 2.23, 0.47–10.62; *P*=0.317), postoperative complications (OR 1.17, 0.59–2.31; *P *= 0.647), dysphagia (OR 1.28, 0.63–2.75; *P *= 0.491), reflux (OR 0.65, 0.29–1.47; *P *= 0.295), and gas bloat syndrome (OR 1.01, 0.45–2.27; *P *= 0.977) were found. However, short gastric vessel division was associated with longer operative duration (MD 25.6min, 95% CI 14.18–37.05; *P* < 0.001) and reduced postoperative lower esophageal sphincter pressure (MD–3.69mmHg, 95% CI −4.11 to −3.26; *P* < 0.001). The overall risk of bias for the two included studies was low (Table 2). In 2018, Kinsey-Trotman et al. reported late results of an RCT with 20-year postoperative outcomes for patients (*n *= 102) undergoing Nissen fundoplication with and without division of short gastric vessels. The groups had no significant difference in heartburn symptoms and satisfaction scores. However, patients who underwent a Nissen fundoplication with a division of the short gastric vessels did have gastric bloating as a symptom more often than patients without a division of short gastric vessels (50% vs. 26%, *P *= 0.046) [22].

### Conclusion

Division of the short gastric vessels does not affect the postoperative symptomatic outcome but prolongs operative time. However, partial dissection of the topmost short gastric vessels might facilitate a tension-free fundoplication and ease the technical aspect of the procedure.

### Recommendation (Level of evidence 1a)

The division of short gastric vessels may be omitted if the fundus can be wrapped around the esophagus without tension.

## Preservation of the vagus nerves

Data were limited to a systematic review assessing the effect of vagus nerve injury on antireflux surgery and one RCT assessing the effect of preservation of the hepatic branch of the anterior vagus nerve. Another retrospective study evaluated the effect of vagotomy on esophageal lengthening. The systematic review by van Rijn et al. included five prospective and three retrospective non-RCTs of poor methodological quality. Therefore, no quantitative synthesis could be performed in the study. Five hundred fifty-nine patients with an intact vagus nerve were compared to 331 patients with vagal nerve injury or dissection. Diarrhea, dumping syndrome, delayed gastric emptying, and gas bloating were less common in patients with intact vagal nerves than in patients with dissected or injured vagal nerves [23].

Ozdogan et al. randomized 40 patients undergoing minimally invasive Nissen fundoplication based on preservation or dissection of the hepatic branch of the vagus nerve. Assessed outcome parameters were fasting gallbladder volumes (25.7 ± 10.0 ml vs. 28.0 ± 12.3 ml; *P *= 0.6) and gallbladder ejection fraction (96.5 ± 1.1% vs. 96.3 ± 1.2%; *P *= 0.6), which were found to be comparable. Gallbladder emptying time, though asymptomatic, was prolonged when the nerve was divided (24.7 ± 8.9 min vs. 34.7 ± 14.4 min; *P *= 0.022) [24]. The authors concluded a higher long-term risk of cholecystolithiasis.

For the study by Ozdogan et al., a low risk of bias was assessed for random sequence generation and allocation concealment. However, patients, personnel, and those assessing outcomes were not blinded by the intervention. Furthermore, the study was not registered in a clinical trial registry. This study, therefore, poses a high risk of performance, detection, and selective reporting bias. Additionally, a large portion of patients were lost to follow-up. Finally, since the study did not specify why the follow-up was incomplete, there was an unclear risk of attrition bias (Table 4).

**Table 4:** Risk of bias assessed for the included randomized controlled trials

Watson 2001	+	+	+	+	+	+	+
Wijnhoven 2008	**+**	**+**	**-**	**+**	**+**	**+**	**-**
Chew 2011	**+**	**+**	**-**	**-**	**+**	**+**	**-**
Ozdogan 2013	**+**	**+**	**-**	**-**	**?**	**-**	**-**
Müller-Stich 2015	**+**	**+**	**+**	**+**	**+**	**+**	**-**
Ilyashenko 2018	**+**	**+**	**-**	**-**	**+**	**+**	**-**
Kinsey-Trotman 2018	**+**	**+**	**+**	**+**	**+**	**+**	**+**
Oor 2018	**+**	**+**	**-**	**+**	**+**	**+**	**-**
Li 2019	**+**	**+**	**-**	**+**	**+**	**-**	**-**
Hopkins 2020	**+**	**+**	**+**	**+**	**+**	**-**	**+**
Watson 2020	**+**	**+**	**-**	**+**	**+**	**+**	**-**
Analatos 2022	**+**	**+**	**+**	**+**	**+**	**+**	**-**
Lang 2023	**+**	**+**	**-**	**+**	**+**	**-**	**-**
	Random sequence generation (selection bias)	Allocation concealment (selection bias)	Blinding of participants and personnel	Blinding of outcome assessment	Incomplete outcome data	Selective reporting	Other bias

+, low risk of bias; -, high risk of bias; ?, unclear risk of bias

A vagotomy has been described as a benign esophagus lengthening procedure in a retrospective analysis of a prospectively maintained database by Oelschlager et al. [25]. The study evaluated symptom improvement in 102 patients who underwent either reoperative antireflux surgery or paraesophageal hernia repair. There were 30 patients with vagotomy and 72 patients without vagotomy in their primary surgery. The study did not show a higher rate of DGE, dumping syndrome, or other side effects in patients after vagotomy compared to the ones without vagotomy during their primary surgery.

### Conclusion

It seems reasonable to preserve the vagal nerve due to the risk of postoperative diarrhea, dumping, and gas bloating, as well as the long-term risk of cholecystolithiasis. However, a selective vagotomy may be helpful for the mobilization of a short esophagus.

### Recommendation (Level of evidence 3b)

Care should be taken care to preserve the vagus nerve during HH repair.

## Cruroplasty

No studies compared different cruroplasty techniques in symptomatic and large HH repair. However, Watson et al. performed an RCT on 102 patients undergoing minimally invasive Nissen fundoplication with either anterior or posterior cruroplasty for anti-reflux surgery. By 6 months after surgery, no difference was found in terms of postoperative dysphagia score [anterior: 5.1 (3.3–6.9) vs. posterior: 6.7 (4.0–9.4); *P* > 0.05], lower esophageal sphincter pressure [25.0mmHg (20.7–29.3) vs. 21.5mmHg (17.6–25.4); *P *= 0.15], heartburn score [0.4 (0.0–0.7) vs. 0.2 (0.1–0.5); *P *= 0.79], and overall satisfaction score [9.0 (8.6–9.4) vs. 8.3 (7.6–9.0); *P* > 0.05]. However, to achieve a similar dysphagia rate, more patients with a posterior cruroplasty had to undergo a second surgical procedure (0% vs. 15%; *P *= 0.03) [26]. At the 5-year follow-up, dysphagia score [8.69 (1.36) vs. 11.20 (1.55); *P *= 0.335] and patient satisfaction score [8.1 (0.41) vs. 7.8 (0.46); *P *= 0.919] were similar. The heartburn score [0.71 (0.22) vs. 1.71 (0.38); *P *= 0.052] tended to be elevated after a posterior cruroplasty, and more reoperations were performed in the posterior group to achieve these results (*n *= 2 vs. *n *= 11; *P *= 0.011) [27]. At the 10-year follow-up, overall dysphagia score [8.95 (6.15–11.76) vs. 11.21 (7.5–14.85); *P *= 0.55] were comparable, apart from solid food dysphagia, which was higher after posterior cruroplasty. The heartburn score [0.70 (0.16–1.23) vs. 1.02 (0.49–1.56); *P *= 0.37] and patient satisfaction score [8.67 (8.02–9.33) vs. 8.07 (7.21–8.93; *P *= 0.43] were similar [28]. According to the Cochrane Collaboration risk-of-bias tool, there was a low risk of bias for all assessed items (Table 3).

### Conclusion

An exclusively posterior hiatoplasty increases the risk of postoperative dysphagia and the associated need for reoperation. It seems essential to avoid creating a hiatal siphon leading to a deviation and narrowing of the esophagus, which might occur if an exclusively posterior cruroplasty is performed.

### Recommendation (Level of evidence 1b)

An exclusively posterior cruroplasty is not recommended since it leads to higher rates of dysphagia postoperatively.

## Addition of a fundoplication

A recent RCT randomized 40 patients with HH plus paraesophageal involvement to mesh-augmented hiatoplasty with cardiophrenicopexy (LMAH-C) or mesh-augmented hiatoplasty with fundoplication (LMAH-F) groups [29]. At 3 months post-op, LMAH-C was associated with a higher DeMeester score (40.9 ± 39.9 vs. 9.6 ± 17; *P *= 0.048). In line with this finding, the authors also observed higher reflux syndrome scores at 12 months in the LMAH-C group (1.9 ± 1.2 vs. 1.1 ± 0.4; *P *= 0.020) and a higher postoperative esophagitis rate (53% vs. 17%) in patients without fundoplication (*P *= 0.026). Dysphagia score (2.1 ± 1.6 vs. 1.9 ± 1.4; *P *= 0.737), postoperative complications (3/20 vs. 1/20; *P *= 0.292), and quality of life (116.0 ± 16.2 vs. 115.9 ± 15.8; *P *= 0.992) were comparable [29]. According to the Cochrane Collaboration risk-of-bias tool, there was a low risk of bias for all assessed items. However, a power calculation was not performed before conducting the trial (Table 3).

Another RCT by Li et al. [30] compared the postoperative outcomes in patients with gastroesophageal reflux disease undergoing isolated minimally invasive HH repair (HHR) with patients (*n *= 122) after minimally invasive HH repair with concomitant Nissen fundoplication (HHR-LNF). After 6 months, an objective reflux assessment based on esophageal manometry and 24-h pH manometry showed a significantly lower DeMeester score in the HHR-LNF compared to the HHR group (7.7 ± 6.8 vs. 12.7 ± 10.1, *P *= 0.017). In addition, a postoperative gastroscopy was performed 12 months after surgery. It showed fewer cases of esophagitis in HHR-LNF than in the HHR group (13.8% vs. 44.6%, *P* < 0.0001). Postoperative patient satisfaction was also assessed on 12-month follow-up after surgery. Most HHR-LNF patients were fully or partially satisfied with the symptom relief following surgery in contrast to patients after HHR (81.8% vs. 47.2%, *P* < 0.001). Clapp et al. performed MA-RCTs and non-RCTs that also favored that additional fundoplication should be routinely performed [31].

### Conclusion

Adding a fundoplication to the repair of large HH leads to less postoperative reflux and esophagitis, irrespective of the evidence of preoperative reflux. Furthermore, it must be taken into account that a reliable assessment of preoperative reflux frequently cannot be provided due to anatomical reasons.

### Recommendation (Level of evidence 2b)

An added fundoplication following the repair of symptomatic and large HH is recommended.

## Type of fundoplication

Multiple RCTs comparing different types of fundoplication for the treatment of gastroesophageal reflux disease resulted in 8 meta-analyses. Four of these meta-analyses compared a partial posterior (270°) with total posterior fundoplication (360°) [32–35]. Two meta-analyses compared an anterior (90°, 120°, 180°) with a posterior (180–360°) fundoplication [36, 37]. One meta-analysis compared partial anterior (180°) with total (360°) and any type of partial (90°, 180°, 200°, 270°, 300°) with total (360°) fundoplication [38, 39]. One meta-analysis of RCTs and non-RCTs compared Nissen fundoplication with less conventional transthoracic Belsey Mark IV fundoplication [40]. The results of these studies are summarized in Table 2. When comparing the different meta-analyses, partial posterior fundoplication (270°) was found to result in less dysphagia and a need for fewer reoperations without impacting heartburn, postoperative complications, and esophagitis rates. Partial anterior fundoplication (180°) was associated with a reduced dysphagia rate, with no differences in the other parameters. Analyses comparing any form of anterior (90°, 120°, 180°) with any form of posterior (180–360°) fundoplication found reduced dysphagia rates in favor of the anterior approach; however, this was at the cost of higher heartburn rates and a tendency for more reoperations. When comparing any form of partial (90°, 180°, 200°, 270°, 300°) to total (360°) fundoplication, total fundoplication had higher dysphagia rates and lower heartburn rates. An RCT published by Hopkins et al. reported on 10-year follow-up in patients who underwent either partial anterior (90°) fundoplication or total (Nissen) fundoplication [41]. Patients after partial anterior reported less dysphagia than patients after total fundoplication (2.03 vs. 3.18 score, *P *= 0.037). However, the patients more often described heartburn symptoms after partial anterior fundoplication (2.83 vs. 1.90 scores, *P *= 0.035).

Analatos et al. compared the outcomes after total Nissen fundoplication (*n *= 32) with partial posterior (200°) fundoplication (*n *= 38) in HH repair in an RCT. While both groups showed significant improvement in dysphagia symptoms and short-term quality of life improvement, the reduction of obstructive symptoms and the long-term quality of life improvement were statistically improved in the group with partial posterior fundoplication [42].

These studies had a low risk of bias, as they clearly stated the main question, were unlikely to have missed important studies, used appropriate criteria to select articles, included sufficiently valid studies, and showed that results were similar from study to study (Table 2).

### Conclusion

Compared to total fundoplication, partial fundoplication is associated with less postoperative dysphagia in the short-term course. However, it has to be kept in mind that both partial posterior fundoplication and an appropriate 180° anterior fundoplication are demanding procedures that should only be performed by surgeons well-trained with these methods. Furthermore, evidence on the long-term effect of partial fundoplication compared to total fundoplication is poor.

### Recommendation (Level of evidence 1a)

Partial posterior fundoplication is the preferred antireflux procedure and, in particular, superior to an anterior fundoplication when added to minimally invasive HH repair. However, total fundoplication might be less technically complex and, therefore, be the procedure of choice for most surgeons.

## Mesh augmentation

Two meta-analyses of RCTs, six meta-analyses of RCTs and non-RCTs, as well as two additional RCTs, were available. Tam et al. included both RCTs and non-RCTs and showed a reduction in recurrence after mesh augmentation (OR 0.51, 0.3–0.87; *P *= 0.014) but no reduction in reoperations (OR 0.42, 0.13–1.37; *P *= 0.149) [43]. Zhang et al. likewise included both RCTs and non-RCTs and confirmed that the overall recurrence rate was lower when a mesh was used (2.6 vs. 9.4%, OR 0.23 (95% CI 0.14–0.39); *P* < 0.001). Furthermore, there was no difference in postoperative complications, but the quality of life following mesh augmentation was found to be higher when compared to that of simple suture repair (MD 13.68, 95% CI 2.51–24.85; *P *= 0.020) [44].

One of the recent meta-analysis of RCTs and non-RCTs by Sathasivam et al. confirmed the reduced recurrence rate with application of mesh augmentation (OR 0.48, 95% CI 0.32–0.73; *P* < 0.05), while the reoperation rate (OR 0.35, 95%CI 0.09–1.31; *P *= 0.12) and complication rate (OR 1.30, 95% CI 0.74–2.29; *P *= 0.36) were comparable [45]. Rajkomar et al. compared HH repair with suture to HH repair with mesh in a meta-analysis of RCTs. They reported a significantly lower total recurrence rate with mesh (OR 0.44, 95% CI 0.25–0.80, *P *= 0.007). However, they mentioned that mesh-augmented HH repair did not reduce the recurrence of HH larger than 2 cm (OR 0.94, 95% CI 0.52–1.67, *P *= 0.83) or the reoperation rate (OR 0.64, 95% CI 0.39–1.07, *P *= 0.09) [46]. Similar results were reported by Temperly et al. in a meta-analysis of RCTs, stating that non-absorbable mesh-augmented HH repair resulted in a lower recurrence rate compared to the suture repair [47]. Clapp et al. report in a meta-analysis of RCTs and non-RCTs as well in favor of a lower recurrence rate after bioabsorbable mesh augmented HH repair compared to the suture alone HH repair [48].

Meta-analyses of RCTs only displayed further advantages of mesh placement. Antoniou et al. examined three RCTs with 267 patients, comparing mesh-augmented HH repair with primary sutures. The recurrence rate was reduced after mesh augmentation at a follow-up between 6 and 12 months (5.8% vs. 24.3%; OR 4.2, 1.8–9.5; *P *= 0.001) [49]. The most recent meta-analysis of five RCTs from Memon et al. confirmed the previously described decreased reoperation rate in patients after mesh augmentation compared to those after primary suture repair [50]. A meta- and risk-benefit analysis underlined the importance of routine mesh placement to repair large (>5 cm) HH. In 915 patients with a 3-year follow-up, mesh augmentation dramatically decreased recurrence rates (20.5% vs. 12.1%, *P *= 0.04). This finding corresponded to an absolute risk reduction for recurrences of 8.4% and a number needed to treat (NNT) of 12 (95% CI, 10.6–13.5). In a subgroup analysis limited to studies with a follow-up longer than 2 years, the reduction of recurrences was even more prominent (25.4% vs. 11.5%; *P *= 0.007). These findings were associated with a decreased risk for reoperations with an absolute risk reduction of 5.6% and an NNT of 18 (95% CI, 13.3–27.3). The mesh-associated complication rate was low (1.9%), and mesh-associated complications did not lead to a higher procedure-related complication rate (OR 1.02, 0.63–1.65; *P *= 0.94) [51]. Ilyashenko et al. performed an RCT comparing patients undergoing large HH repair with mesh augmentation to those undergoing large HH repair with suture cruroplasty. They used nonabsorbable sutures for cruroplasty and a ProGrip mesh of which the size and shape were individually determined depending on the anatomical hiatal surface areas (HSAs). Only patients with HSAs between 10 and 20 cm^2^ were included in the study. The ProGrip mesh did not require sutures for fixation. During the 48 months of follow-up, one recurrence occurred in the mesh group and eight recurrences appeared in the non-mesh group (*P *= 0.027). Patient satisfaction was significantly higher in the mesh group (*P *= 0.004). They concluded that reinforcing crura with mesh is safe and can prevent recurrences after large HH repair [52].

In the meta- and risk/benefit-analysis by Müller-Stich et al., polypropylene was the most used mesh (39.6%), associated with a low complication rate of 0.8%. Polytetrafluorethylene (31.9%) and biologic meshes (13.5%) were also widely used but with higher associated complication rates of 2.5% and 1.3%, respectively. A recent meta-analysis revealed a reduced short-term recurrence rate for biologic mesh compared to primary sutures (3.5% vs. 16.6%; OR 3.74, 95% CI 1.55–8.98, *P *= 0.003). However, 42% of biologic mesh cases and 51% of primary suture repairs experienced long-term HH recurrence (OR 1.43, 95 % CI 0.56–3.63, *P *= 0.45), demonstrating that biologic meshes are less effective than permanent meshes in terms of prevention of long-term recurrence [53].

On the other hand, Oor et al. compared 72 patients with large HH undergoing HH surgical repair with either nonabsorbable sutures or nonabsorbable sutures reinforced with non-absorbable U-shaped mesh. The RCT did not show a significant difference regarding the postoperative 6-month HH recurrence rate between the two groups, questioning the necessity of mesh augmentation (14.3% vs. 17.2%, *P *= 0.746). However, the RCT by Oor et al. is limited to 36 patients in each group and can thus be considered underpowered and should therefore be interpreted with caution [54]. Similarly to Oor et al. a meta-analysis by Campos et al. showed no significant differences in favor of any of the intervention methods (mesh versus suture cruroplasty) for large HH repair the different outcomes evaluated: recurrence (RD −0.06, CI [−0.13,0.01], *I*^2^ 22%, *p* 0.27); postoperative complications (RD 0.04, CI [−0.01,0.9], *I*^2^ 5%, *p* 0.30); deaths (RD −0.01, CI [−0.04, 0.02], *I*^2^ 0%, *p* 74); intraoperative complications (RD −0.03, CI [−0.07, 0.1]); reoperation (RD −0.04, CI [−0.10, 0.02], *p* 0.14) [55].

An RCT has been published with 5-year follow-ups comparing minimally invasive repair sutures of very large HH, absorbable, and non-absorbable 2–3 cm × 4–5 cm mesh posterior repair anchored with sutures or tacks mesh with no difference in recurrence rate and outcomes [56, 57]. Similar results have been reported in two meta-analyses of RCTs describing comparable early and late recurrence rates between both HH repair with sutures and mesh-augmented. However, these studies have yet to distinguish between large and non-large HH [58, 59].

The studies on routine mesh augmentation and mesh material had a low risk of bias, as they clearly stated the main question, were unlikely to have missed important studies, used appropriate criteria to select articles, included sufficiently valid studies, and showed that results were similar from study to study (Table 2).

No prospective or retrospective studies have compared different forms of mesh fixation at the hiatus. However, several reports of life-threatening and fatal complications have been reported when using tacks for mesh fixation to the diaphragm [60–62]. Therefore, tacks should not be used for mesh fixation at the diaphragm, and fibrin glue may be a safer alternative.

### Conclusion

Mesh augmentation reduces the risk of recurrence and the associated need for reoperation after repairing large HH (>5cm) with paraesophageal involvement, while the overall complication rate is unaffected. Therefore, the benefits outweigh the risk of mesh-related complications, especially considering the complexity of reoperations after HH repair. When a synthetic mesh is used, a quickly integrating material such as polypropylene should be used, as polytetrafluorethylene has been associated with inferior tissue integration characteristics. Mesh fixation with fibrin glue may be preferable to other forms of mesh fixation. Tacks should be avoided.

### Recommendation (Level of evidence 1a)

Routine use of mesh augmentation is recommended for the repair of large HH (>5cm) with paraesophageal involvement to reduce the risk of recurrence and the need for a potentially complex reoperation.

### Recommendation (Level of evidence 2b)

Due to their superior long-term effects, synthetic meshes should be favored over biological meshes.

### Recommendation (Level of evidence 4)

Mesh fixation with fibrin glue may be preferable to other forms of mesh fixation, at least in the anterior tendinous part of the diaphragm, to avoid potentially fatal complications.

## Discussion

This systematic review provides recommendations for eight important technical aspects of surgical repair of symptomatic and large HH based on the highest level of evidence available. To guarantee that these recommendations have been determined accurately, the systematic review grouped the included studies according to the technical step evaluated and sorted them according to the levels of the Oxford Centre for Evidence-based Medicine. Each question was searched for level 1 to 5 evidence, and only the highest level was considered for the respective recommendation.

Compared to the previous guidelines published by the SAGES Guidelines Committee [1], the evidence mapping presented here emphasizes the need for routine fundoplication and mesh augmentation for large HH. Additionally, the present review searched for evidence on the effect of the dissection of the short gastric vessels, the preservation of the vagus nerve, and the optimal type of fundoplication.

Even though a high level of evidence was available for most topics, the present review also revealed several evidence gaps. For example, no RCTs compared complete dissection of the hernia sac with no dissection. However, such a trial is unlikely to be conducted, as it is accepted practice to completely dissect the hernia sac due to the practical technical benefits. Some of the included studies described higher conversion rates in patients without complete hernia sac dissection. Nevertheless, it is important to mention the potential technical difficulties of the surgical cases, which could be the reason why the dissection was not performed in the first place, and the conversion to an open surgery took place. On the other hand, the dissection of the hernia sac may also facilitate the surgery and may therefore avoid conversions and other complications. The HH recurrence rate was significantly higher in patients without complete hernia sac dissection. Thus, the complete dissection of the hernia sac was listed as a recommendation for symptomatic and large HH repair.

No studies were found comparing the surgical treatment of the HH regarding the choice of performing a division of short gastric vessels. Thus, studies that included patients undergoing antireflux surgery with or without the division of short gastric vessels were evaluated. However, partial transection of the uppermost short gastric vessels during surgical repair of symptomatic and large HH may facilitate tension-free fundoplication.

Moreover, only one RCT with various risks of bias evaluated the effect of preservation of the vagus nerve, and a systematic review assessing the topic could not perform a quantitative synthesis. Nevertheless, the studies included in the systematic review by van Rijn et al. predominantly described that diarrhea, dumping syndrome, delayed gastric emptying, and gas bloating were less common in patients with preserved vagal nerve than in patients with dissected or injured vagal nerve. The endpoints of the one RCT by Ozdogan et al. were postoperative fasting gallbladder volumes, gallbladder ejection fraction, and gallbladder emptying time. It must be mentioned that this RCT specifically evaluated the dissection of the hepatic branch of the vagus nerve. Therefore, it might be considered outside of the scope of the presented study on the optimal surgical repair of symptomatic and large HH. However, the postoperative occurrence of the above-mentioned pathophysiological changes in the gallbladder function, even after dissection of only the hepatic branch of the vagus nerve, was an interesting aspect of the potential postoperative symptoms in patients with postoperatively dissected vagus nerve. Therefore, the RCT by Ozdogan et al. was included in the presented assessment. Thus, to avoid postoperative symptoms related to the dissection of the vagus nerve, it is advised to preserve the vagus nerve whenever possible when performing surgical repair of symptomatic and large HH. Adequately designed studies focusing on symptomatic postoperative outcomes are needed.

Furthermore, there are no studies that investigated in a randomized controlled setting how different types of cruroplasty affect postoperative outcomes and quality of life in patients with large HH repair. Therefore, three RCTs that investigated whether posterior cruroplasty was inferior to other forms of hiatoplasty and whether routine fundoplication was needed for paraesophageal HH repair needed to be included in the analysis. This additionally limits the evidence support in recommendations of the type of cruroplasty in large HH repair since suitable studies have not yet been performed on the matter. Therefore, we have cautiously phrased the recommendations for cruroplasty in the large HH repair and strongly agree that evidence regarding the matter would be bolstered by appropriate high-quality (multicenter) RCTs.

The studies included in the assessment of the use of mesh in symptomatic and large HH repair had a low risk of bias and spoke favorably for the use of mesh. However, several RCTs, such as the RCT by Oor et al., did not show a significant difference regarding postoperative 6-month HH recurrence rate between the patients after HH repair with sutures and patients after HH repair with non-absorbable mesh. Thus, it is important to define the recommendation for when to use the mesh augmentation, and that is when it comes to symptomatic and large HH repair.

The studies on routine mesh augmentation and mesh material had a low risk of bias, as they clearly stated the main question, were unlikely to have missed important studies, used appropriate criteria to select articles, included sufficiently valid studies, and showed that results were similar from study to study (Table 2).

Mesh augmentation reduces the risk of recurrence and the associated need for reoperation after the repair of large HH (>5 cm) with paraesophageal involvement. Simultaneously, the overall complication rate is not affected. Therefore, the benefits can outweigh the risks of mesh-related complications, especially when considering the complexity of reoperations after HH repair.

It is important to mention that the recommendations for division of the short gastric vessels, preservation of the vagus nerve, cruroplasty, and type of fundoplication were based on studies that also included patients with gastroesophageal reflux and small HH since there were no studies that solely included patients with large HH.

Furthermore, robotic-assisted surgery has gained popularity in various surgical specialties, including the repair of large HHs. Repairing these hernias can be challenging, and the use of robotic technology offers both benefits and downsides in this context. The robotic approach to large HH repair offers precision, visualization, and patient recovery advantages. However, it also comes with certain drawbacks, such as cost, a surgeon learning curve, and potential technological limitations. The decision to use robotic-assisted surgery should be made on a case-by-case basis, considering the patient’s specific needs and the surgeon’s expertise. However, the conventional laparoscopic approach is well-established and effective, and there is no evidence to support the preference for robotic-assisted over the conventional laparoscopic approach for large HH repair. Ultimately, the choice between laparoscopy and robotic-assisted surgery for large HH repair will depend on the patient’s specific conditions, the availability of the technology, and the surgeon’s skills and preferences.

To summarize, we propose the following ten statements regarding eight important technical issues of HH repair:

1. MIS offers several benefits over the open approach, such as reduced postoperative morbidity and a shorter hospital stay (*Level of evidence 1a*).
2. Complete dissection and excision of the hernia sac may reduce the risk of conversion, postoperative recurrences, and complications, maybe due to better visualization and mobilization of the esophagus and a tension-free positioning of the stomach within the abdominal cavity (*Level of evidence 3b*).
3. Division of the short gastric vessels does not affect the postoperative symptomatic outcome but prolongs operative time. However, partial dissection of the topmost short gastric vessels might facilitate a tension-free fundoplication (*Level of evidence 1a*).
4. It seems reasonable to preserve the vagal nerve due to the risk of postoperative diarrhea, dumping, and gas bloating and the long-term risk of cholecystolithiasis (*Level of evidence 3b*).
5. A dorsal-ventral hiatoplasty is recommended. An exclusive posterior hiatoplasty increases the risk of postoperative dysphagia and the associated need for reoperation. It seems essential to avoid creating a hiatal siphon leading to a deviation and narrowing of the esophagus, which might be the consequence of an exclusive posterior cruroplasty **(***Level of evidence 1b*).
6. The addition of fundoplication to the repair of large HH leads to less postoperative reflux and esophagitis, irrespective of the evidence of preoperative reflux. Furthermore, it has to be taken into account that a reliable evaluation of preoperative reflux frequently cannot be provided due to anatomical reasons (*Level of evidence 2b*).
7. Compared to total (360°) fundoplication, partial fundoplication is associated with less postoperative dysphagia in the short-term course. However, it must be kept in mind that both partial posterior (180°, 270°) fundoplication and an appropriate anterior (180°) fundoplication are technically complex procedures that should only be performed by surgeons well-trained with these methods. Furthermore, evidence on the long-term effect of partial fundoplication in comparison with total fundoplication is poor (*Level of evidence 1a*).
8. Mesh augmentation reduces the risk of recurrence and the associated need for reoperation after the repair of large HH (>5 cm) with paraesophageal involvement. Simultaneously, the overall complication rate is not affected. Thus, the benefits can outweigh the risk of mesh-related complications, especially when considering the complexity of reoperations after HH repair (*Level of evidence 1a*).

If a synthetic mesh is used, a quick integrating material such as polypropylene should be used, as polytetrafluorethylene has been associated with inferior tissue integration characteristics. Biologic meshes are effectless in the long term (*Level of evidence 2b*). Mesh fixation with fibrin glue may be preferable to other forms of mesh fixation. Tacks or stitches should be avoided, at least in the anterior tendinous half of the hiatus (*Level of evidence 4*).

## Conclusions

Evidence mapping is a reasonable instrument based on the best evidence available to guide surgeons in determining how best to perform an HH repair. According to that, HH repair should be performed in a repair suture approach, include a dorso-ventral cruroplasty, the addition of fundoplication, and, for large HH with paraesophageal involvement, a mesh augmentation should be performed.

### Supplementary information

ESM 1 (DOCX 93 kb)

ESM 2 (DOCX 17 kb)
