# Supplementary material for: Evidence mapping on how to perform an optimal surgical repair of large hiatal hernias
Source: Langenbecks Arch Surg. 2023 Dec 21;409(1):15. doi: 10.1007/s00423-023-03190-y (PMC10733223; doi:10.1007/s00423-023-03190-y)
Supplement: Supplementary file 1 — (DOCX 93 kb) [file 423_2023_3190_MOESM1_ESM.docx]

((((((hiatal[tiab] OR hiatus[tiab]) AND hernia*[tiab]) OR (((gastroesophageal*[tiab] OR gastrooesophageal*[tiab] OR gastro-esophageal*[tiab] OR gastro-oesophageal*[tiab]) AND reflux*[tiab]) OR GERD[tiab])) AND (repair*[tiab] OR laparoscop*[tiab] OR (minimal*[tiab] AND invasiv*[tiab]) OR laparotom*[tiab] OR surger*[tiab] OR surgical[tiab] OR (open[tiab] AND (treatment[tiab] OR management[tiab] OR procedure*[tiab])) OR herniorrhaph*[tiab])) OR ((("Herniorrhaphy"[Mesh] AND ((hiatal[tiab] OR hiatus[tiab]) AND hernia*[tiab]))) OR "Gastroesophageal Reflux/surgery"[Mesh] OR "Hernia, Hiatal/surgery"[Mesh])) OR ((((hiatal[tiab] OR hiatus[tiab]) AND hernia*[tiab]) OR "Hernia, Hiatal"[Mesh] OR ((gastroesophageal*[tiab] OR gastrooesophageal*[tiab] OR gastro-esophageal*[tiab] OR gastro-oesophageal*[tiab]) AND reflux[tiab]) OR GERD[tiab] OR "Gastroesophageal Reflux"[Mesh]) AND (("short gastric vessels"[tiab] OR "short gastric vessel"[tiab] OR "short gastric arteries"[tiab] OR "short gastric artery"[tiab] OR "Arteriae gastricae breves"[tiab] OR "Splenic Artery"[Mesh] OR (fundus[tiab] AND (mobilization*[tiab] OR mobilize*[tiab]))) OR (cruroplast*[tiab] OR cruroraph*[tiab]) OR (fundoplicatio*[tiab] OR "Fundoplication"[Mesh] OR "NISSEN procedure"[tiab] OR "NISSEN surgery"[tiab]) OR (sac[tiab] AND (excision*[tiab] OR resection*[tiab] OR dissection*[tiab])) OR ((nerv*[tiab] AND (vagus*[tiab] OR vagal*[tiab])) OR (vagal[tiab] AND injur*[tiab]) OR "Vagus Nerve"[Mesh]) OR (((mesh*[tiab] OR "Surgical Mesh"[Mesh]) AND (material*[tiab] OR fixation*[tiab] OR augmentation[tiab] OR placement[tiab] OR "reinforced repair"[tiab] OR "mesh-reinforced repair"[tiab])))))) NOT (animals[mh] NOT humans[mh])
