# Supplementary material for: Evidence mapping on how to perform an optimal surgical repair of large hiatal hernias
Source: Langenbecks Arch Surg. 2023 Dec 21;409(1):15. doi: 10.1007/s00423-023-03190-y (PMC10733223; doi:10.1007/s00423-023-03190-y)
Supplement: Supplementary file 2 — (DOCX 17 kb) [file 423_2023_3190_MOESM2_ESM.docx]

**Table 5:** Meta-analyses comparing different forms of fundoplication.

|  |  | **II Included studies** | **Dysphagia** | **Heartburn** | **Esophagitis** | **Reoperation** | **Complications** |
| --- | --- | --- | --- | --- | --- | --- | --- |
| **Partial posterior (270°) vs. total (360°) posterior** | | | | | | | |
| Broeders et al., 2010 | | 7 | RR 1.61  (1.06, 2.44)  P=0.02 | RR 1.11  (0.75, 1.63)  P=0.61 | RR 1.20  (0.78, 1.85)  P=0.40 | RR 2.19  (1.09, 4.40)  P= 0.03 | RR 0.62  (0.29, 1.32)  P=0.22 |
| Tan et al., 2010 | | 7 | OR 2.81  (1.52, 5.22)  P=0.001 | OR 1.01  (0.48, 2.13)  P=0.97 | n.a. | n.a. | OR 0.31  (0.11, 0.93)  P=0.04 |
| Tian et al., 2015 | | 13 | RR 2.61  (1.76, 3.87)  P<0.01 | LNF 14%  LTF 12%  P = 0.45 | n.a. | LNF 4.74%  LTF 6.54%  P= 0.77 | RR 0.67  (0.39, 1.14)  P= 0.14 |
| Du et al., 2016 | | 8 | RR = 2.75  (1.69, 4.50)  P <0.001 | RR = 0.88  (0.48, 1.64)  P=0.70 | RR = 0.98  (0.81, 1.18) P=0.80 | RR = 3.16  (1.49, 6.68)  P=0.003 | RR 0.78  (0.37, 1.68)  P=0.53 |
| **Anterior (90°, 120°, 180°) vs. total (360°) posterior** | | | | | | | |
| Broeders et al., 2011 | | 7 | WMD -2.87  (-3.88, -1.87)  P<0.001 | RR 2.71  (1.72, 4.26)  P= 0.001 | RR 2.17  (0.67, 7.02)  P=0.19 | RR 1.94  (0.97, 3.87)  P=0.06 | RR 1.06  (0.54, 2.08)  P=0.86 |
| Memon et al., 2014 | | 9 | SMD -2.31  (-3.19, -1.43)  P= 0.001 | OR 1.90  (1.04, 3.49)  P=0.037 | n.a. | OR 1.66  (0.91, 3.04)  P=0.10 | OR 1.07  (0.45, 2.53)  P= 0.88 |
| **Partial (180°) anterior vs. total (360°) posterior** | | | | | | | |
| Broeders et al., 2013 | | 5 | WMD -2.25  (-2.66, -1.83)  P<0.001 | SMD 1.27  (-0.36, 2.90)  P= 0.13 | RR 1.42  (0.69, 2.91)  P= 0.34 | RR 2.08  (0.80, 5.41)  P=0.13 | RR 2.18  (0.69, 6.93)  P=0.19 |
| **Partial (90°, 180°, 200°, 270°, 300°) vs. total (360°)** | | | | | | | |
| Ma et al., 2010 | | 13 | OR 0.44  (0.30, 0.62)  P< 0.0001 | OR 1.94  (1.18, 3.18)  P<0.01 | n.a. | n.a. | n.a. |
| Values are presented as RR, OR, WMD, SMD (95% confidence interval). *P<0.05; **P<0.01; n.a., not available; OR, odds ratio; RR, relative risk; WMD, weighted mean difference; SMD, standardized mean difference. | | | | | | | |
